# First Use of Non-Invasive Spinal Cord Stimulation in Motor Rehabilitation of Children with Spinal Muscular Atrophy

**DOI:** 10.3390/life13020449

**Published:** 2023-02-05

**Authors:** Anton Novikov, Maria Maldova, Natalia Shandybina, Ivan Shalmiev, Elena Shoshina, Natalia Epoyan, Tatiana Moshonkina

**Affiliations:** 1 EirMED, 10 Vsevolod Vishnevsky St., 197136 St. Petersburg, Russia; 2 Pavlov Institute of Physiology, Russian Academy of Sciences, 6 Makarova Enb., 199034 St. Petersburg, Russia; 3 Almazov National Medical Research Centre of the Ministry of Health of the Russian Federation, 2 Akkuratova St., 197341 St. Petersburg, Russia

**Keywords:** spinal muscular atrophy, spinal cord stimulation, physical therapy, nusinersen

## Abstract

Spinal muscular atrophy (SMA) is characterized by the degeneration of spinal alpha motorneurons. Nusinersen demonstrated good efficacy in the early disease phases. The feasibility of transcutaneous spinal cord stimulation (tSCS) in motor rehabilitation of patients with spinal cord injury has been demonstrated. We hypothesize that tSCS may activate intact and restored by nusinersen motorneurons and slow down the decline in motor activity, and may contribute to the development of motor skills in children with SMA. A case series is presented. Five children (6–13 years old) with SMA type II or III participated in the study. They were treated with nusinersen for ~2 years. Application of tSCS was carried out during physical therapy for 30–40 min per day in the course of 10–14 days. Outcome measures were goniometry of joints with contracture, forced vital capacity (FVC), RULM and HFMSE scales. The participants tolerated the stimulation well. The reduction of the contracture was ≥5 deg. RULM and HFMSE increased by ~1–2 points. Predicted FVC increased by 1–7% in three participants. Each participant expanded their range of active movements and/or learned new motor skills. Spinal cord stimulation may be an effective rehabilitation method in patients treated with nusinersen. More research is needed.

## 1. Introduction

Spinal muscular atrophy (SMA) is a genetic disease, with an estimated prevalence of ~1:100,000 [1]. SMA is characterized by the degeneration of alpha motor neurons in the anterior horns of the spinal cord, leading to progressive weakness and atrophy of the proximal muscles [2]. Three main SMA phenotypes differ in the age of onset and the maximum achievable motor function associated with this age. Type I manifests in the first 6 months after birth, the maximum motor capabilities are sitting with support. Type II—between 6 and 18 first months of life, patients with type II are capable of sitting independently. Type III—after 18 months of life, patients are capable of standing upright and walking. In real practice, phenotypes are a continuum rather than distinct types. Clinicians identify additional subtypes, and SMA patients are described by functional status as non-sitting, sitting, and walking [3]. Rehabilitation of patients with SMA is aimed at slowing down the process of motor skill loss, improving the quality of life, and reducing disease burden. It has been shown that physical therapy may influence the trajectories of SMA progression [3].

SMA is caused by deletion or mutation of the SMN1 gene—a gene responsible for activity and survival of motor neurons. The paralogous SMN2 gene is similar to the SMN1 gene, except for a few single nucleotide exchanges, including one in exon 7 leading to aberrant pre-mRNA splicing that results in the skipping of exon 7 in almost 90% of the transcripts. SMA severity is correlated as with SMN2 gene copies carried by patients [1]. The first disease-modifying drug, nusinersen, was approved by regulatory authorities for the pharmaceutical market, and showed good efficacy in the early disease phases [4]. Nusinersen activity is based on the correction of exon 7 splicing of the endogenous SMN2 pre-mRNA. Expectations have arisen that now, since the first approved medication for the treatment of patients with SMA has become available, a fundamental change in the natural course of the disease can be achieved if optimal care is combined with effective treatments [5].

The efficiency and safety of using electrical transcutaneous spinal cord stimulation (tSCS) in motor rehabilitation of the adult and pediatric patients with severe spinal cord injury have been demonstrated [6,7,8,9,10]. Additionally, tSCS is being used in the rehabilitation of children with cerebral palsy [11,12]. In [11], the experimental group received tSCS at 2 levels, above the T11 and L2 vertebrae, in combination with locomotor training on a treadmill, while the control group received similar training without stimulation. After stimulation, an increase in torque in the knee joint was observed in the patients of the experimental group, while this effect was absent in the patients of the control group. In addition, a decrease in the coactivation of the muscles of the lower extremities was observed in the patients of the experimental group. Thus, lower extremity muscle strength increased and joint stiffness, correlated with muscle coactivation [13], decreased after tSCS combined with motor training. Since maintaining muscle strength and preventing the development of joint contracture is the goal of motor rehabilitation in SMA [3], we would argue that spinal stimulation combined with motor training may be useful in SMA patients’ rehabilitation.

For non-invasive electrical stimulation of the spinal cord, rectangular bipolar or monopolar pulses modulated at a frequency of 5–10 kHz are used. This complex shape makes it possible to increase the strength of the current to excite the afferents of the posterior roots of the spinal cord and thereby modulate the motor neurons activity [14]. The tSCS is well tolerated by healthy subjects with normal sensitivity [15], by pediatric patients with spinal injury [12] and with cerebral palsy [11]. In last two years, it has been revealed that the kilohertz carrier frequency does not affect pain tolerance and the posterior radicular motor evoked responses in adult healthy subjects [16,17]. Thus, the significance of the carrier frequency in the effects of tSCS is called into question. With regard to these new data, it should be taken into account that the 5 kHz carrier frequency contributed to the cortical inhibitory effects as was shown in tetraplegic patients as well as in healthy subjects [18]. The arm function in tetraplegic patients improved largely when tSCS was used with the 5 kHz carrier frequency compared to tSCS used without it [18].

In all the aforementioned cases where tSCS has been used to activate spinal locomotor centers located in “healthy” areas of the spinal cord, motor deficits were caused by signal disruptions between the brain and musculature or abnormal brain activity. In SMA, spinal alpha motor neurons are genetically affected, muscle weakness progresses, and then muscle atrophy develops as a result. It has recently been shown that after treatment with nusinersen over the course of 18 months, the number of motor neurons increases dramatically [19]. We hypothesized that in children with SMA, the stimulation can activate intact motor neurons and motor neurons that have been restored after nusinersen treatment, resulting in a deceleration in the decline in motor activity and the development of motor skills.

## 2. Materials and Methods

All procedures, training and testings were performed in the period between August and October 2022 in the EirMED rehabilitation center (St. Petersburg, Russia). The procedures and investigations were performed in accordance with the Declaration of Helsinki and approved by the Ethics Committee of EirMED Rehabilitation Center (#22-01, date of approval 12 August 2022). Five children with SMA (6–13 years) with genetically confirmed type II or III SMA participated in the study (Table 1). Participants had a functional status of either “non-sitter” or “sitter” [3] and individual orthoses were used for each one. All children were treated with Spinraza™ (nusinersen) for approximately 2 years. They had previously received regular physical therapy aimed at preventing loss of muscle strength, development of joint contractures, and scoliosis.

Spinal cord stimulation was used simultaneously with physical therapy. These interventions were aimed at achieving a personalized rehabilitation goal (Table 2). The goals of the treatment were individualized and determined by the SMART (specific, measurable, achievable, realistic/relevant and timed) method because this way was an effective method of achieving a behavioral change [20].

The application of tSCS was carried out as an individualized intervention aimed at reducing joint contracture (except participant P146 who had no contractures), increasing physical endurance, and teaching motor skills to improve quality of life (Table 3). Physical therapy consisted of passive and active stretching movements in the joints of the upper and lower extremities; passive and active positioning; moving and holding weights; body movements to prevent scoliosis; stepping and kicking movements, and breathing exercises. In some cases, in order to facilitate movements of the lower extremities, the movements occurred in the gravity-neutral position of the legs, when the participant was lying on their side, with the legs supported by a swing [15].

One or two regions of the spinal cord were stimulated, above the cervical or lumbar enlargement of the spinal cord and/or above the cauda equina of the spinal cord, as previously described [6,7,8,9,11,12,15]. When exposed to the muscles of the upper extremities, stimulation of the neck and upper thoracic region was used. To influence the muscles of the trunk and lower extremities, stimulation of the lower thoracic, lumbar and coccygeal regions was used. Two adhesive round electrodes (Ø 2.5 cm) were placed on the skin above the spinous process of either of C7, Th11, L1, and L5 vertebrae or coccyx (Table 3). These electrodes were independent cathodes. Two adhesive rectangular electrodes (4 × 5 cm^2^) above iliac crests were common anodes. Stimulation sites were selected depending on the rehabilitation goals, considering the results of all previously published studies [8,10,11,21,22]. Bipolar rectangular pulses of 1 ms duration, 20 Hz (for P146 participant) or 30 Hz (for other participants), modulated with 5 kHz, produced by Neostim-5 (Cosyma Ltd., Moscow, Russia). The intensity of stimulation has been determined for each participant individually so that the current amplitude was maximally tolerable, without any unpleasant sensations. In the case of participant P146, tSCS appeared uncomfortable when the intensity was lowered to 10 mA, so the frequency was reduced while the stimulation intensity was kept within the range used for the other participants.

Stimulation was carried out 6 days a week and lasted for 30–40 min, excluding breaks to relax participants and change positions for the next therapeutic exercise. Physical therapy with tSCS took ~1 h per day.

Moreover, physical procedures were carried out to improve coordination and proprioception, such as massage, and balneotherapy. In addition, there were sessions with an ergo therapist and a speech therapist. The intensity of those sessions was adapted to the individual constitution of the participants. All therapies including training and procedures took 3 h per day. In total, the duration of therapy was 10–14 days for each participant.

Outcome measures were specific for pediatric SMA patients [23]. These methods are aimed at measuring muscle strength, motor functions, and respiration. Efficacy criteria were goniometry of joints with contracture [24], Revised Upper Limb Module (RULM) [25], and modified Hammersmith Function Motor Scale Expanded (HFMSE) [26] when available.

Following standards of goniometry [24], a single determination of the range of passive movements in the knee and elbow joints was carried out. The authors of this guide postulated that averaged measure was no more reproducible than single measurements of the joint angles. Goniometry of the knee was performed in the prone position. Range of the elbow motion was tested in supine.

RULM is a robust clinical measure to assess upper limb motor function in SMA [27]. It is a 20-item scale with a maximum score of 37. The scale establishes functional levels covering distal to proximal movements. It was appropriate for all participants.

HFMSE is a standard scale of functional ability in children with SMA types 2 and 3 but it is not appropriate to non-sitters [26]. This scale comprises 33 items; the maximum possible score is 66.

Forced vital capacity (FVC) has also been tested [28], as it has been found to be strongly correlated with motor function [29] and appears to be the most reliable measure among other pulmonary parameters to use as outcomes in SMA [23]. By [28], absolute values of the FVC were determined three times. The largest FVC observed from three of the acceptable values was compared with age-matched controls as a percent predicted for age, which is calculated on the basis of height.

Testing was carried out the day before and the next day after the therapeutic course in the absence of tSCS.

Adverse events of tSCS were monitored daily. The risk level of the study is minimal since the tSCS procedure is non-invasive. No adverse reactions or adverse events were detected when this stimulation was tested in pediatric patients [8,9,11].

Trainers and parents of participants were interviewed on the last day of the course about significant changes in motor activity and motor skills that they observed during the course of the study. Trainers documented each procedure, so they reported changes in the duration of positions and holding time.

## 3. Results

The participants tolerated the stimulation well. No adverse events were observed.

**Figure 1:**
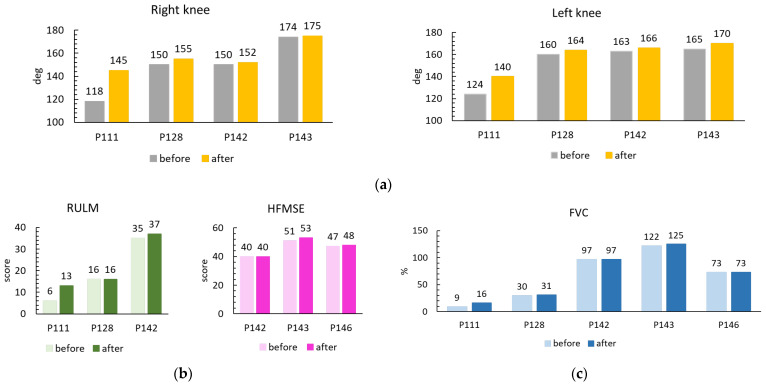
Measured outcomes demonstrating the results of the course of tSCS in combination with physical therapy in participants with SMA treated with nusinersen. Each column represents one measurement according to the standards for each of the tests used. The tests were carried out the day before and the next day after the treatment course. (**a**) Goniometry of joints with contracture. (**b**) Results of upper extremities motor function (RULM) and functional ability (HFMSE) scaling. (**c**) Forced vital capacity, percentage predicted based on height and age.

Four of five participants had contractures of knee joints (Figure 1a). After rehabilitation, the joint shortening was reduced by 1–5 deg in P128, P142, and P143 participants. In non-sitter P111, contracture of the knee joints reduced at 16 and 27 deg. The last participant had the elbow joints contracture and reduction of those contractures was achieved as well: the angles in the right elbow before/after the course were 134/146 deg, in the left—137/141 deg, respectively.

Treating upper limb disability is critical to optimizing day-to-day functions for non-sitters. Participant P111’s score on the RULM scale doubled from 6 points (Figure 1b) and included a new motor skill—she learned to bring a plastic cup of water to her mouth. This skill was observed both by researchers during testing and by parents in everyday life (Table 3). Participants P143 and P146 had maximal RULM scores (37 points) before and after tSCS; therefore, their results are not included in Figure 1b. The increase in RULM scores for other participants was limited. The trainers and parents of non-sitters focused on increasing the range of voluntary movements in the shoulder and elbow joints (Table 4). Participant P143 began to hold a load of 0.5 kg with a lateral arm abduction for up to half a minute.

HFMSE, a scale of the functional ability in individuals with SMA, is not available to non-sitters (participants P111 and P128). Testing of HFMSF of sitters P142, P143 and P146 showed an increase of 0, 2 and 1 points, respectively (Figure 1b). However, functionality not included in the HFMSE changed in four participants. The length of sitting independently of participant P128 increased from 20 s to 3 min; participant P142 learned to move from the couch to the wheelchair, from the wheelchair to the floor and back to the couch, and managed to pull himself to a standing position while holding on to the bars of the Swedish Wall; the time of independent vertical posture of P143 increased by three times; participant P146 learned to sit down from a supine position, pulling himself up on his hands (Supplement, Video S1) (Table 4).

FVC increased by 7%, 1% and 3% of predicted values based on height and age in participants P111, P143 and P146, respectively, and was not changed in others (Figure 1c). An increase in FVC indicates an increase in respiratory muscle strength in these participants.

## 4. Discussion

This study analyzed the efficacy and safety of spinal cord stimulation used in the movement rehabilitation of SMA patients treated with nusinersen. In our cohort of five participants, we did not observe any adverse events, providing initial safety data for the use of tSCS in pediatric SMA patients.

We show that a 2-week physical therapy protocol combining tSCS with the use of nusinersen led to a decrease in contracture of up to 27 deg in one of our participants. In all participants with permanent shortening of muscles or joints (n = 4), the reduction of the contracture was ≥5 deg in one of the joints. Thus, the main goal of the rehabilitation of sitters and non-sitters—prevention of contractures, and joint stretching [3]—was achieved.

The increase in the results on the RULM and HFMSE scales after the course does not seem impressive. On the other hand, the level of minimal clinically important differences (the smallest improvement that the patient considers worthwhile) in the scales are not available to pediatric SMA patients so far [30]. Investigations on the effect of nusinersen on adult SMA patients revealed a significant improvement by two points in RULM and ~1 point in HFMSE when compared with untreated patients after a six month period [31]. Therefore, a 1–2 point change of RULM and HFMSE may be important for SMA patients. In the phase 3 trial of nusinersen in children with SMA II older than 6 years (similar to the participants in our investigation), the increase of HFMSE was ≤2 points after 15 months of drug treatment (Figure 2A in [32]). Thus, the increase in motor activity after two weeks of rehabilitation instead of >1 year in the participant P143 (13 years old) and participant P146 (7 years old), potentially saved significant time (Figure 1b). The goal of rehabilitation for non-sitters and sitters pediatric patients with SMA is to slow the progression of the disease, not to improve motor functions. Therefore, the positive results demonstrated here represent a marked improvement over standard of care [3].

The small increase in FVC results obtained after tSCS treatment is similar to the small increase observed in RULM and HFMSE scores. In patients with SMA, an increase in FVC is strongly correlated with an increase in muscle strength [29] and with the increase of HFMSE [30]. The maximum increase of 7% of normal values from the initial value of 16% was recorded in participant P111, who demonstrated the maximum increase in the RULM score in this group (from 6 to 13). As previously stated, the results of the RULM scale correlate with muscle strength. Participants P128 and P143 showed increases in FVC of 1% and 3% of normal values, respectively, and these results are compared to no change in RULM scores and moderate increases in HFMSE scores in these participants. Participants that did not show increases in FVC demonstrated small increase in RULM (participant P142) and in HFMSE (participant P146).

It is interesting to note that participant P111, who could not bring a plastic cup of water weighing 0.2 kg to her mouth prior to the study and who became capable of doing it after the course completion (Table 4), was able to move and lift a load of two times greater mass than a cup of water while the exercise therapy in combination with tSCS was in progress (Table 3). A similar strengthening effect was observed in previous studies of tSCS in motor rehabilitation of patients with spinal cord injury when tSCS increased muscle strength. During stimulation, patients were able to stand independently and move their lower limbs voluntarily, but could not perform these tasks without stimulation [6]. Subjects with spinal cord injury at the cervical level were investigated with a water bottle test half an hour after 20 min of tSCS or sham-tSCS, applied between C5 and C6 spinous processes [18]. For the water bottle test, subjects were asked to lift a 6 cm diameter bottle, filled with 200 mL of water, from the table and pour the water into a cup. The test execution time after tSCS was 50% of the time after sham-tSCS. Thus, we observed a movement-facilitating effect of tSCS in participant P111, similar to that of tSCS in people with spinal cord injury. This is indirect evidence that the effect of tSCS on the motor system of SMA patients is similar to that of individuals with spinal cord injury.

In all previous studies, when tSCS was used for rehabilitation [6,9], it transynaptically activated preserved spinal motor neurons. Presumably in SMA patients, spinal stimulation increased the activity of damaged and consecutively restored by nusinersen motor neurons. At the same time, the motor improvement induced by tSCS may be due to the effect on spinal cord neural networks rather than a direct effect on motor neurons. Studies in spinalized rats have shown that the recovery of locomotion after spinal cord stimulation and locomotor training correlates with the ordering of interneuron activity in the lumbar segments and a decrease in their excessive activity, which was observed in the absence of supraspinal influences [33,34]. A recent review [35] discusses data showing that motor dysfunction in SMA is not limited to motor neurons, but manifests itself in spinal networks in which motor neurons are embedded. In addition, motor impairment in SMA patients correlates with structural abnormalities in the skeletal muscles [29] and electrical stimulation induces muscle fiber transformation. Muscle structure transformation after neuromuscular stimulation is well known [36], yet nothing is known about the effect of spinal cord stimulation on the morphology and histochemistry of the muscles. Thus, the causes and mechanisms of the resulting improvement in the motor activity of pediatric patients with SMA after a course of motor therapy combined with tSCS require further investigation.

Clinical studies of the effect of physical therapy combined with nusinersen treatment on motor activity have not been conducted previously, and we find the comparison of our findings with other results difficult; one cannot single out the role of tSCS itself in the resulting motor improvements. The absence of a control group as well as a short period of observation are limitations of our analysis. The study presented is a case series which could lay a foundation for further studies, including randomized controlled trials [37]. Our preliminary results are important as we demonstrated that the combination of optimal drug therapy with tSCS and physical therapy over a short period of only 2 weeks resulted in improved motor function in every participant. The goals of rehabilitation of non-sitters and sitters SMA patients are optimizing function, optimizing tolerance to various positions, preventing contractures, and restoring mobility. Each participant expanded their range of voluntary movements and/or learned new motor skills, making daily life easier for patients and their families. In the last two years, many articles on the use of tSCS in studies appear and many researchers have used this method for motor rehabilitation. We believe it is essential to attract their attention to the restoration of patients with SMA because medication for the treatment of spinal motor neuron disease is approved and, potentially, spinal cord stimulation may be affected by these motor neurons.

## 5. Conclusions

A course of non-invasive electrical spinal cord stimulation combined with physical therapy in children with SMA treated with nusinersen resulted in a reduction in contractures, an increase in muscle strength and forced lung capacity, each participant expanded the range of active movements and/or mastered new motor skills. The course lasted 2 weeks, and the results are comparable with the results that are obtained after a course of nusinersen lasting more than a year. Participants tolerated the intervention well. This impact can be an effective method of rehabilitation for SMA. More research is needed.

## Figures and Tables

**Table 1:** Participant demographics and clinical parameters.

PCPs	Sex	Age (Years)	SMA Type	SMN2 Copy NUMBER	Starting Nusinersen	Functional Status
P111	F	9	II	3	October 2020	non-sitter
P128	M	6	II	3	April 2021	non-sitter
P142	M	10	III	3	November 2020	sitter
P143	M	13	III	4	September 2020	sitter
P146	M	7	III	3	January 2020	sitter

**Table 2:** Personalized rehabilitation goals.

PCPs	Rehabilitation Goal
P111	reduction of contractures of the knee and elbow joints; increasing the accuracy and coordination of movements of the upper extremities; increasing of the range of active movements in the joints of the lower extremities
P128	reduction of contractures of the knee joints; independent sitting; increasing of the muscle strength in the upper extremities; increasing of the range of active movements in the joints of the lower extremities
P142	reduction of contractures of the knee joints; independent sitting; increasing of the muscle strength in the shoulders; decreasing of hypertonicity in the buttocks; supported standing position
P143	reduction of contractures of the knee joints; increasing of the muscle strength in the right shoulder; increasing of the muscle strength in the trunk; independent standing position
P146	increasing of the muscle strength in the trunk; increasing of the muscle strength in the hips; independent standing position; independent symmetrical stepping

**Table 3:** Stimulation parameters and training details.

PCPs	tSCS			Physical Therapy		
	Site (Vert)	Intensity (mA)	Trainings (N)	Upper Extremities	Lower Extremities ^1^	Other
P111	Th11, Co	20	12	grasping, passive flexion/extension of the elbow joints, passive adduction/abduction of the shoulder joints, passive stretching arms above the head	flexion/extension of the toes, *passive flexion/extension of the knee joints, stepping, kicking*	holding and moving objects ~0.5 kg, long expiration breath
P128	C7-Th1, Co	30	10	grasping, flexion/extension of the elbow joints, adduction/abduction of the shoulder joints, stretching arms above the head, ball kicking and hit accuracy training	*flexion/extension of the knee joints, stepping, kicking*	holding and moving objects ~1 kg, long expiration breath, trunk twists and tilts
P142	C7, L1	50	14	flexion/extension of the elbow joints, adduction/abduction of the shoulder joints, stretching arms above the head, ball throwing	flexion/extension of the knee joints, adduction/abduction of the hip joints, stepping for 2 min, kicking	holding and moving objects ~2 kg, trunk twists and tilts
P143	C7, Th12	15	12	flexion/extension of the elbow joints, adduction/abduction of the shoulder joints, stretching arms above the head, ball throwing	flexion/extension of the knee joints, adduction/abduction of the hip joints, stepping for 2 min, kicking	holding and moving objects ~2 kg, trunk twists and tilts
P146	C7, L1	27	12	stretching arms above the head, lifting arms on the sides, ball throwing	stepping for 5 min, ball kicking	holding and moving objects ~0.5 kg, trunk twists and tilts

^1^*in italics*—training in the gravity-neutral position of the legs.

**Table 4:** Opinion of the trainers and parents about significant changes in motor activity and acquisition of new skills during the course of tSCS in combination with physical therapy.

PCPs	Upper Extremities	Lower Extremities	Other
P111	increased range of active motion in all joints; can bring a plastic cup of water to her mouth	increased range of active motion in all joints	
P128	active abduction in the shoulder joints of both arms increased		sitting without support increased from 20 s to 3 min
P142			was able to transfer from the couch to the wheelchair, from the wheelchair to the floor, from the floor to the couch without help; to pull himself up to standing position holding on the Swedish Wall and remain standing with support for up to 11 s
P143	the time of holding a load weighing 0.5 kg with the lateral abduction of the arm increased from 0 to 30 s		the time of upright posture without support increased from 3 to 10 s
P146			rise from lying on the back position to a sitting position pulling himself up holding a movable support ^1^

^1^Video S1, Supplement.

## Data Availability

The datasets generated during and/or analyzed during the current study are available from the corresponding author on reasonable request.

## Supplementary Materials

The following supporting information can be downloaded at: https://www.mdpi.com/article/10.3390/life13020449/s1; Video S1: P146.mp4.
